# Penetration study of p-methoxycinnamic acid (PMCA) in nanostructured lipid carrier, solid lipid nanoparticles, and simple cream into the rat skin

**DOI:** 10.1038/s41598-022-23514-0

**Published:** 2022-11-12

**Authors:** Noorma Rosita, Angguni Addini Sultani, Dewi Melani Hariyadi

**Affiliations:** Department of Pharmaceutical Sciences, Faculty of Pharmacy, Universitas Airlangga, Surabaya, Indonesia

**Keywords:** Medical research, Rheumatology

## Abstract

This study compared the ability of Nanostructured Lipid Carrier (NLC), Solid Lipid Nanoparticles (SLN), and Cream systems in delivering para Methoxycinnamic Acid (PMCA) to the dermis layer of the skin. Wistar rats were used as research subjects. NLC and SLN were made by applying the high shear homogenization method. Nile red was used as a penetration indicator based on its fluorescence. The interaction between fluorescence labeled NLC, SLN, or Cream and rat skin was visualized by fluorescence microscopy. Observations were made after 2 and 4.5 h of smearing the test sample. From the observations, it was known that the system/lipid base could penetrate the stratum corneum for delivering drugs. Penetration speed differs among systems as does the number of PMCAs that can be delivered. In this study, it can be concluded that the NLC system is able to deliver PMCA more quickly and in greater quantities to the dermis than SLN and Cream.

## Introduction

Topical preparation is a drug preparation in which its administration system is intended to be used on the skin^1^. The advantage of topical preparation is that it can avoid first-pass metabolism, reduce side effects caused by oral use, and improve patient compliance^2^.

On its development, conventional topical preparation has a problem, namely low permeation^3^ or low uptake due to barrier function of the stratum corneum^5^, thus a proper delivery system right to the place of action is needed. One of them is Lipid nanoparticle system. Lipid nanoparticle system is a semi-solid emulsion system with nano-size particles, which can be made by the application of high shear homogenization or high pressure homogenization method.

This drug delivery system is characterized by its matrix components consisting of single solid lipid or mixed lipid^6^. According to Khezri et al. (2018), topical preparation containing lipid nanoparticle has advantages, such as increasing the penetration of active substances into the skin so that it can increase effectiveness. The occlusive nature of this system enhances the hydration effect and the skin elasticity due to the small size of the lipid (nanoparticle) can form a lipid layer above the skin surface so that more water in the skin is retained^4^. This causes a reduction in the corneocyte wrapping, therefore the size of the corneocyte gap increases, and then more drugs will be absorbed^4^. There are two generations of Lipid nanoparticle, namely Solid Lipid Nanoparticle (SLN) and Nanostructured Lipid Carriers (NLC)^5^. According to Patel et al. (2019), both are safe as drug carriers via the topical route. SLN is made by replacing the oil from the o/w emulsion with solid lipid that is safe for the body^8^. Meanwhile, NLC has a composition consisting of a mixture of solid lipid and liquid lipid. NLC which its composition consists of a mixture of solid and liquid lipids can cause the formation of an imperfect lipid matrix so that the drug is loaded more^9^. The difference in composition between SLN and NLC causes NLC to have an advantage over SLN, which is higher drug entrapment but lower active ingredient pressure, so that the stability of NLC is better than SLN^3,9^. The choice of composition between solid lipid and liquid lipid in the NLC system is an important factor in drug entrapment that will affect drug release and penetration. In addition, with the nanoparticle size and spherical shape that NLC has, it can increase drug penetration into the skin^11^ and will produce a fast penetration rate at the beginning. NLC with a high entrapment value will produce a slow but constant penetration rate. Topical drug targeting the dermis must be able to penetrate the stratum corneum in which the drug will then interact with the target receptor. For this reason, it is necessary to carry out a penetration test.

This research used p- Methoxycinnamic Acid (PMCA) as the active agent with NLC delivery system. APMS is a biologically active compound resulting from the hydrolysis of Ethyl p- Methoxycinnamate (EPMS) derived from the Kaempferia galanga or aromatic ginger^12^. PMCA penetration is affected by the stratum corneum^12^, so that for the PMCA formulation it is necessary to add enhancers, or choose a drug delivery system that can affect the stratum corneum, such as NLC. In this study, the lipid components used are cetyl alcohol and oleic acid. These types of lipids affect the character of SLN or NLC. According to the research conducted^12^, cetyl alcohol can produce SLN with a smaller size and more spherical shape than stearic acid and can increase drug entrapment. Oleic acid as a liquid lipid can increase in vivo Trans Epidermal Water Loss (TEWL) and transport the drug to the subcutaneous intercellular area^15^. In the research done by Hu^16^, NLC with solid lipid stearic acid and oleic acid (liquid lipid) shows faster drug release than NLC without oleic acid.

In this research, penetration test of PMCA will be carried out with the ratio of NLC composition used of cetyl alcohol and oleic acid is (85%: 15%) of the total lipid in the formula. This was based on a research^16^, which states the addition of oleic acid up to 15% increases drug release with almost the same particle size distribution. The addition of liquid lipid will indirectly affect the absorption of the drug into the skin, thus it affects the penetration of the drug into the skin. In the SLN and NLC systems, it was reported that the controlled release caused by the burst released of the drug can increased the penetration of the drug into the skin^10^.

The increasing of PMCA penetration in NLC was proven by comparing it with SLN system and simple cream. To find out how the penetration effectiveness of the NLC system in the skin, the fluorescent label included in the formula was used and observed using a fluorescent microscope. The fluorescent label used was nile red, which is lipophilic^17^.

## Results and discussion

### Particle size

The results of the particle size examination (Table 1) showed that all formulas were included within the nanoparticle size range, which was 10-500nm^18^ and obtained the particle size of NLC PMCA < SLN PMCA < PMCA cream. This was because there was a liquid lipid in the NLC PMCA formula, namely oleic acid which could help the solubility of PMCA so that the resulting particle size was smaller.

**Table 1:** Particle size and polydispersity index (PI) of PMCA cream, PMCA_SLN, NLC base, and PMCA_NLC.

Formula	Particle size (nm)	PI ± SD	% CV
PMCA Cream	207.5	–	–
PMCA_SLN	136.6	0.506 ± 0.03	0.03
NLC Base	65.2	0.280 ± 0.04	0.04
PMCA_NLC	89.5	0.369 ± 0.01	0.01

From the PI values showed that the NLC base was most homogeneous compared to others, while PMCA_NLC was more homogeneous than PMCA_SLN. This might be caused by the addition of liquid lipids in the PMCA_NLC that dissolve PMCA.

### pH formula measurement

The pH of the APMS, SLN APMS, and NLC APMS cream formulas in Table 2 in accordance with the pH skin range of 4–6^19^.

**Table 2:** pH of PMCA cream, PMCA_SLN, NLC base, and PMCA_NLC.

Formula	pH ± SD	% CV
PMCA Cream	3.97 ± 0.03	0.8
PMCA_SLN	4.01 ± 0.01	0.14
NLC Base	4.13 ± 0.01	0.24
PMCA_NLC	4.07 ± 0.03	0.75

### Entrapment efficiency (EE)

Previously, a homogeneity test was performed to determine the uniformity of levels carried out for each replication conducted. Based on the results of the recovery test, it was known that the recovery of each repeatability formula was good because the recovery value of each formula met the requirements, namely 85–115%^20^. In addition, this method was considered to have good repeatability because of the small % CV.

**Figure 1:**
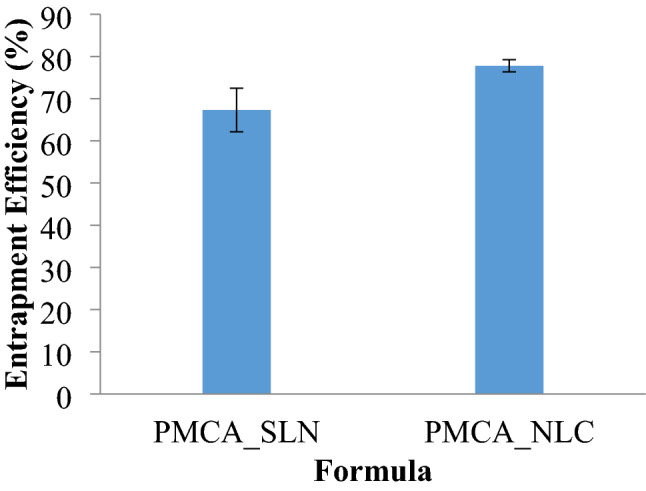
Entrapment efficiency of PMCA_SLN and PMCA_NLC.

From the results of the determination of EE, obtained data that can be seen in Fig. 1. The NLC PMCA formula had greater entrapment efficiency. The addition of liquid lipids in the formula affected the imperfections of the crystal lattice so that during solidification of the lipid phase, many drugs were trapped in the system^21^. Besides, it can also leave enough space for drug placement and drug entrapment^16^.

The data were analysed statistically with T-test sample method, it obtained the value of α sig. 2 tailed counts (0.028) < 0.05. It indicated that there was a significant difference and the Entrapment Efficiency of PMCA_NLC was greater than PMCA_SLN.

### Occlusivity

**Figure 2:**
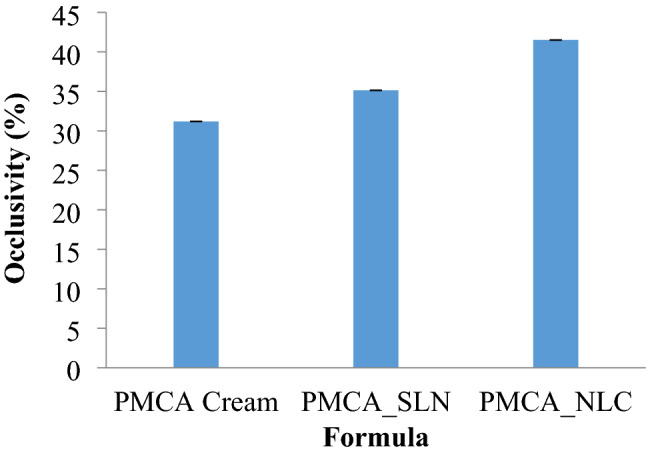
Occlusivity of PMCA cream PMCA_SLN, and PMCA_NLC.

The results of the occlusivity test of each formula is provided in histogram of the average occlusivity for one week from the various formulas which can be seen in Fig. 2. PMCA_NLC had greater occlusivity than PMCA_SLN and PMCA_Cream. This was because PMCA_NLC had the smallest particle size and the most homogen particle size. Small particle size could cause the structure between particles to be tight, causing an increased occlusivity, and small particle size caused the surface area of particles in contact with the skin to increase^22^. The presence of occlusivity caused the skin hydration increased. Moreover, hydration of the stratum corneum affected the rate of substance penetration into the skin.

The occlusivity data were analyzed using one-way Annova statistics. A calculated α value (0.00) < 0.05 indicated that there was a significant difference. Then proceed with the HSD Tukey test. The results showed there was a significant difference between the formulas. In this study, the largest occlusiveness was PMCA_NLC.

### Penetration test on rat skin

**Figure 3:**
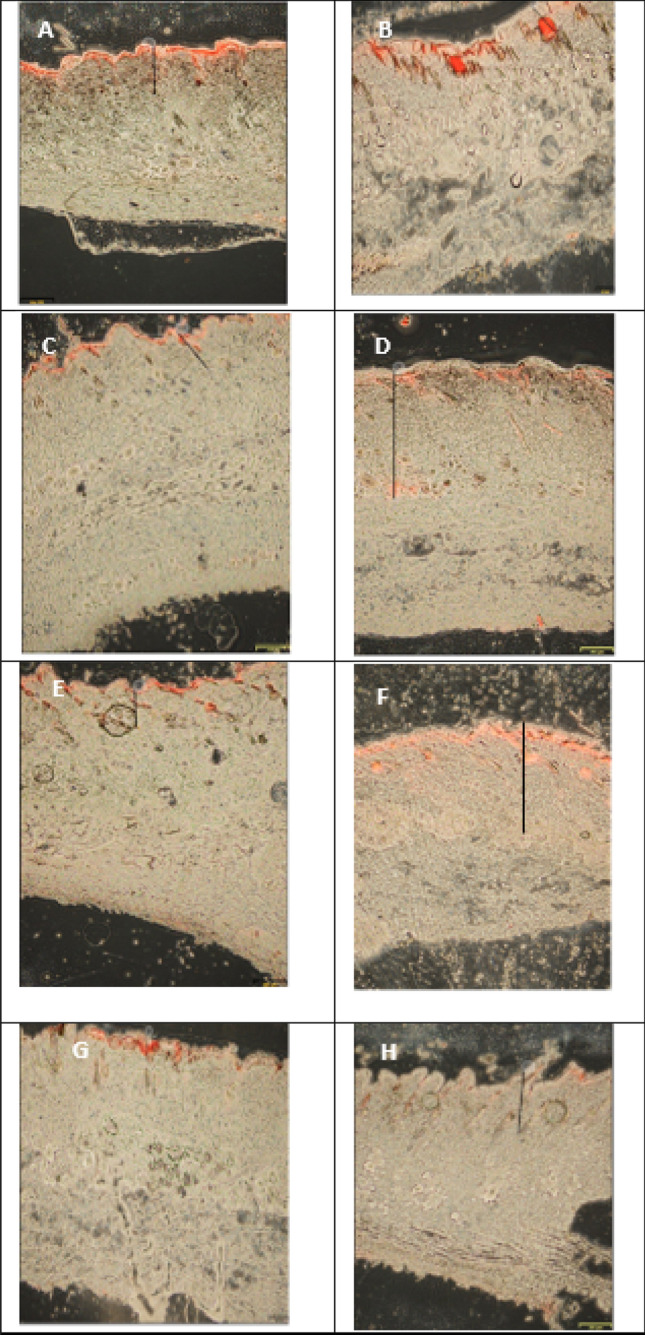
Penetration and distribution of nile red in rat skin of PMCA cream, observed after 2 h (**A**) and 4.5 h (**B**), PMCA_SLN observed after 2 h (**C**) and 4.5 h (**D**), NLC base observed after 2 h (**E**) and 4.5 h (**F**), PMCA_NLC observed after 2 h (**G**) and 4.5 h (**H**).

**Figure 4:**
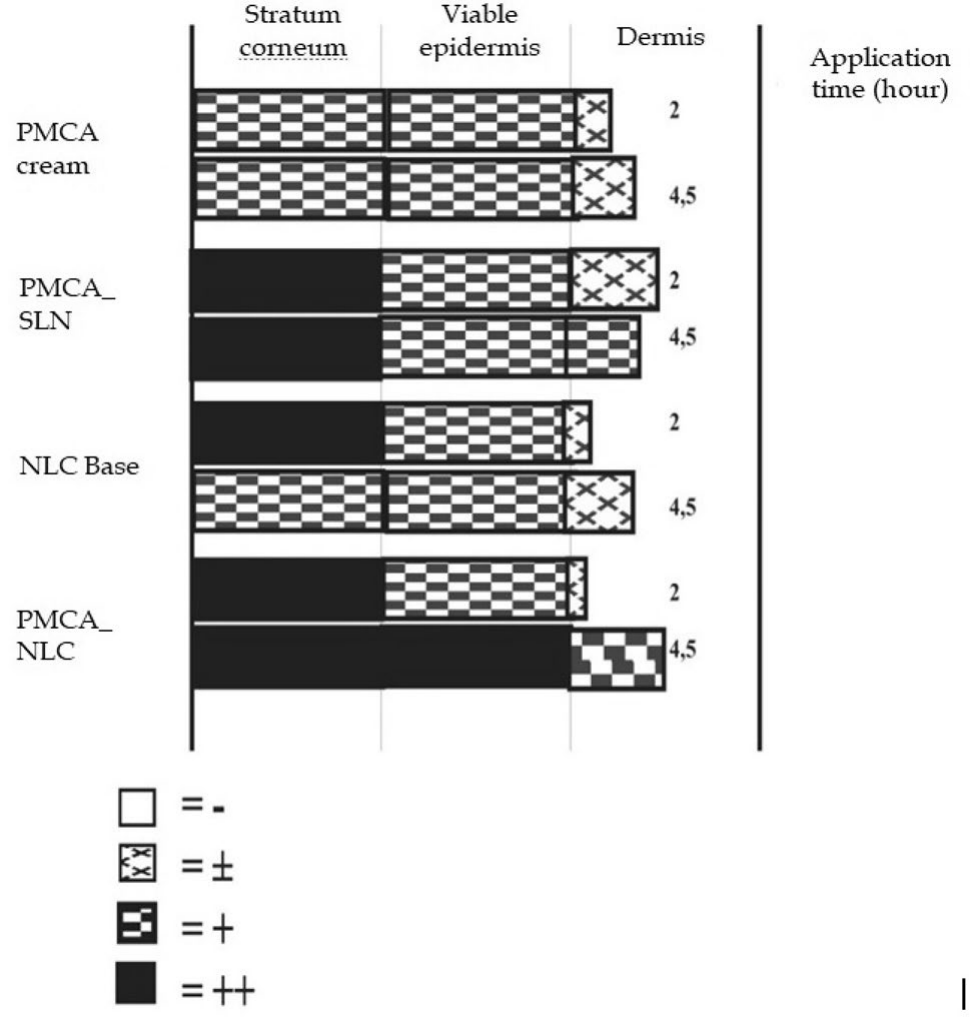
Intensity of fluorescence of PMCA cream, PMCA_SLN, NLC base and PMCA_NLC after the 2nd and 4.5th hour of application.

On the penetration test, nile red was used as a fluorescent label because of its lipophilic nature^23^, otherwise known as lipophilic dye. As can be seen in Figs. 3 and 4, the NLC base in the first 2 h penetrated deeply into the dermis layer. This proved that the NLC base penetrated deeply into the skin layer, as well as PMCA in cream, SLN and NLC. This was because Cream, SLN, and NLC are lipid-base colloid carriers. Lipid-containing carrier system is a good drug delivery system for the skin because it can facilitate penetration, act on the skin surface, repair adhesiveness, and increase the hydration of corneocytes in the stratum corneum^9^.

The depth of penetration of the three formulas were compared over time (Fig. 4 and Table 3). After the application for 2 h, PMCA SLN penetrated deeper than PMCA NLC and PMCA Cream. This was predicted to occur due to the effect of differences of lipid crystal modification in SLN and NLC. Crystal modification causes less drug entrapment in SLN than in NLC, so that the amount of drug in the outer phase of the SLN was more than that in the NLC. This result has also happened in a research^24^ which indicates that the crystalline modification of the SLN matrix forces the drug out to the outer phase and accelerates penetration. Meanwhile, after the application for 4.5 h, NLC PMCA penetrated deeper compared to PMCA SLN and PMCA Cream. The results were different with the 2 h result. These differences could be caused by various factors that affect drug penetration into the skin such as occlusivity, particle size, drug concentration, and the effect of carrier materials^25^. The difference in PMCA NLC and PMCA Cream was more influenced by particle size. The small particle size and the spherical shape of the particles cause the inter-particle structure to be tight, causing an increased occlusivity (water retained in the stratum corneum caused hydration and swelling of corneocyte cells in the stratum corneum layer), so that drug substances could enter easily^26^. The difference between PMCA NLC and PMCA SLN was due to the addition of oleic acid as a liquid lipid, which also has other functions, namely as a penetration enhancer^25^ and PMCA solubilizing agent. The function of oleic acid as a penetration enhancer is to increase the drug transport to the subcutaneous intercellular area^15^. The increase of solubility of PMCA increased the diffusion coefficient PMCA to the skin membrane so that the drug penetrated better^26^. The fluorescence intensity measured qualitatively by observing the amount of drug delivered by Cream, SLN, and NLC to the skin layers, namely the stratum corneum, viable epidermis, and dermis. PMCA NLC could carry more drugs than PMCA SLN and PMCA Cream (Fig. 4.). Up to 4.5 h, the PMCA in the NLC system still had a fairly clear intensity red luminescence of nile in the dermis layer.

**Table 3:** Depth of penetration into the rat skin after application for 2 and 4.5 h.

Formula	Depth of penetration (µm) into the rat skin ± SD (% CV) after application for:		
		2 h	4.5 h
PMCA Cream		507.93 ± 102.9 (20%)	288.63 ± 112.64 (39%)
PMCA_SLN		996.23 ± 638.00 (64%)	799.14 ± 228.89 (29%)
NLC	Base	200.65 ± 103.95 (52%)	531.44 ± 332.96 (63%)
	PMCA	412.82 ± 90.72 (22%)	1079.19 ± 152.26 (14%)

## Methods and materials

The materials used in this study were p-methoxycinnamic acid (PMCA) from sigma Aldrich, cetyl alcohol, oleic acid, Tween 80 and propylene glycol from PT Brataco, Nile red from sigma Aldrich, isopropyl myristate (IPM), glacial acetic acid and sodium acetate with grades of quality from Merck index, and the distilled water from PT. Brataco.

### Selection and treatment of animals

The experimental animals selected as research subjects were male white rats with Wistar strain (Rattus norvegicus). Male rats were selected to avoid the hormonal influence of the female. The rats used were 2–3 months old and weighted 200–300 g. The procedure for treating experimental animals in this study had received approval from the Research Ethics Commission of the Faculty of Veterinary Medicine, Universitas Airlangga (Certificate number 2.KE.016.02.2020). All methods were performed in accordance with ARRIVE guidelines and the relevant guidelines and regulations respectively.

### Sample preparation

The test preparations in this study were PMCA NLC, NLC-Base, PMCA SLN, and PMCA Cream. PMCA NLC, NLC-Base, and PMCA SLN were made by applying high shear homogenization using Ultra Turrax T-25 at 24,000 rpm for 8 min in 4 cycles. At the same time, the manufacture of PMCA cream was made in a conventional way.

## Characterization of SLN, NLC, and Cream

### Particle size

The determination of particle size was carried out using the DelsaTM Nano Particle Analyzer. The data taken from this instrument was the average diameter of the particle size and polydisperty index.

### pH measurement

The pH value of the preparation was measured using a pH meter by dispersing 1 g of sample into 10 mL of aquademineralisata.

### Entrapment efficiency (EE)

The determination of EE in this study used the dialysis method. A portion of the dialysis media was taken and then its absorption was measured at the maximum wavelength of PMCA with a UV–Vis spectrophotometer to determine the amount of PMCA that was not trapped in the NLC. The blank used was the same NLC system with each formula without the addition of PMCA and prepared as same as the test sample. After that, it was calculated using the formula:

$$ {\text{EP}}\left( \% \right) \, = \, \left[ {\left( {{\text{Ct}} - {\text{Cf}}} \right)/{\text{Ct}}} \right] \, \times { 1}00\% $$

Notes:

Ct: PMCA concentration in NLC

Cf: PMCA concentration that are not entrapped

### Viscosity

The viscosity of the preparation was measured using a Brookfield Cone and Plate HDV-I viscometer rate of share 20 rpm at 30 °C.

### Occlusivity test

The occlusivity test was carried out using in vitro method. First of all, the empty vial was weighted and dried. Then, the vial that already contained 5 ml of water was weighted, closed using a filter membrane (cellulose filter, 0.45 mm pore, Whatman no. 4), and tied with a rope. After that, 10 drops of Isopropyl Myristate were added to saturate the membrane and left it overnight. After that, 0.05 g of the preparation to be tested was applied evenly to the membrane and then observed for a week. After a week, the amount of water that had been lost was calculated with the following formula:

$$F=\left(\frac{A-B}{A} \right)x 100$$

Notes:

A = Amount of water lost in control

B = Amount of water lost in sample

F = Occlusivity

### Penetration test on rat skin

On the early-stage skin penetration test, the rats were anesthetized by giving them ketamine of 20 mg/kg BW by intra muscular (im). The hair on the abdomen was shaved using mechanical hair clipper, then 50 mg of the test sample was applied to the stomach evenly. The part that had been smeared with the test sample was covered with a plastic cover and wrapped with a bandage so that not much of the test sample was lost and ensured that the test sample penetrated the skin layer well. After 2 and 4.5 h, the rats were sacrificed by cervical dislocation. The part of the skin that had been smeared with the sample was cut with an area of 1 × 4 cm^2^ then it was put on aluminum foil so it did not shrink and stored in the freezer at −80 °C. The skin that had been cut was then cut again using a frozen microtome with a thickness of 5 m. The finished preparation was then observed using a fluorescent microscope FSX100 OLYMPUS.

Furthermore, a qualitative analysis was carried out on the data obtained, namely the penetration distance of the test sample into the skin layer and the fluorescence intensity in each layer of the skin. Each layer of the skin was assessed for fluorescent intensity, namely: (-) No fluorescence, ( ±) Weak fluorescence, ( +) strong fluorescence, and (+ +) very strong Fluorescence^27^.

## Conclusions

In this study, it can be concluded that the NLC drug delivery system can carry PMCA drugs to penetrate the dermis layer. In addition, the NLC system is able to increase drug penetration (PMCA) deeper compared to SLN and Cream as a delivery system.

## Data Availability

The datasets used and/or analysed during the current study available from the corresponding author on reasonable request.
